# Modulation of Fibroblast Growth Factor 19 Expression by Bile Acids, Meal Replacement and Energy Drinks, Milk, and Coffee

**DOI:** 10.1371/journal.pone.0085558

**Published:** 2014-01-20

**Authors:** Amanda M. Styer, Stephen L. Roesch, George Argyropoulos

**Affiliations:** 1 Weis Center for Research, Geisinger Health System, Danville, Pennsylvania, United States of America; 2 Institute of Obesity, Geisinger Health System, Danville, Pennsylvania, United States of America; University of Bari & Consorzio Mario Negri Sud, Italy

## Abstract

**Background:**

The enterohepatic pathway involving the fibroblast growth factor 19 (FGF19) and bile acids (BA) has been linked with the etiology and remission of type 2 diabetes (T2D) following Roux-en-Y gastric bypass (RYGB) surgery. Specifically, diabetic patients had lower FGF19 circulating levels but postoperative FGF19 and BA levels were higher in diabetic patients that experience remission of T2D, as compared to non-diabetic patients and diabetic patients that do not experience remission. It has been proposed that this may be due to the direct flow of digestate-free bile acids into the ileum benefiting mostly T2D patients without severe diabetes.

**Methods/Results:**

We used a human colorectal cell line (LS174T) that endogenously expresses FGF19, real time PCR, and Elisas for precise quantitation of FGF19 mRNA and secreted protein levels. We report here that BA and fractions of BA stimulated FGF19 *in vitro* but this effect was partially blocked when BA were pre-incubated with a lipoprotein mix which emulates digested food. In addition, we show that FGF19 mRNA was stimulated by meal replacement drinks (Ensure, Glucerna, SlimFast), non-fat milk, and coffee which has been linked with reduced risk for developing diabetes. Pure caffeine and the 5-hour Energy drink, on the other hand, decreased FGF19 mRNA.

**Conclusions:**

In summary, FGF19 expression *in vitro* is modifiable by popular drinks suggesting that such approaches could potentially be used for modulating FGF19 expression in humans.

## Introduction

Fibroblast growth factors regulate processes of development, transformation, and angiogenesis, functioning as hormones [1], [2]. Fibroblast growth factor 19 (FGF19) and FGF21 also share the ability to regulate glucose, lipid, and energy homeostasis [3]. FGF15 (FGF19 in humans) and FGF21 transgenic mice are resistant to diet-induced obesity and have improved insulin sensitivity, glucose disposal, and lipid parameters [4], [5]. Injection of recombinant FGF19 protein into diabetic mice resulted in the reduction of serum glucose, improved glucose tolerance, and body weight [2], [3], [6]. In addition FGF19 reversed dietary diabetes [7] and improved glycogen synthesis in an insulin-independent pathway [8].

In humans, serum levels of FGF19 were increased postprandially by chenodeoxycholic acid (CDCA) and decreased by bile acid sequestrants [9]. This outcome was corroborated in cell culture using the intestinal cell line LS174T, whereby, lithocholic (LTA) and chenodeoxycholic (CDCA) acids were found to potently stimulate FGF19 expression [10] through multiple FXR response elements in its promoter [11]. FGF19 is produced primarily in the ileum and signals in hepatocytes through its two receptors, fibroblast growth factor receptor 4 (FGFR4) and βKlotho, to inhibit expression of cholesterol 7alpha-hydroxylase 1, (*CYP7A1*) [12]. In response to FGF19, *CYP7A1* regulates the rate-controlling step for the conversion of cholesterol into bile acids [13]. By a feedback mechanism, BA also regulate hepatic *CYP7A1* gene expression through a multicomponent pathway involving hepatic FXR [14].

We recently showed that FGF19 levels were lower and bile acids higher in patients with type 2 diabetes (referred to herein as “T2D” or “diabetes”) [15]. In addition, low FGF19 levels were correlated with higher hepatic expression of *CYP7A1* only in diabetic patients, while, FGF19 and bile acids levels were increased at higher rates in diabetic patients that went in remission of T2D after Roux-en-Y gastric bypass (RYGB) surgery, compared to non-diabetic or T2D patient that did not go in diabetes remission [15]. We [15] and others [16] have suggested that “digestate-free” bile acids after RYGB surgery may be more bioactive and thus responsible for the increased stimulation of enteric FGF19. This hypothesis was tested in the present study.

In addition, coffee (caffeinated and decaffeinated) has been associated with reduced risk for type 2 diabetes [17]–[19] through a mechanism that has yet-to-be determined. Similarly, it is not known if meal replacement liquids and popular energy drinks have any effect on FGF19. Here, we set out to determine whether lipoproteins can block the stimulation of FGF19 by bile acids, and examine the effects of popular meal replacements, milk, coffee, and energy drinks on FGF19 expression using the human colorectal LS174T cells.

## Materials and Methods

### Cell Culture and Treatments

The human colorectal adenocarcinoma LS174T cell line was purchased from ATCC (Manassas, VA) and grown in Eagle’s Minimum Essential Medium with 10% fetal bovine serum and 1% Penn/Strep at 37°C and 5% CO_2_.

The unconjugated bile acids cholic, deoxycholic, and chenodeoxycholic acid, as well as the corresponding tauro- and glycine-conjugated bile acids were purchased from Sigma-Aldrich (St Louis, MO), and were used combined (i.e., total BA) or singularly. Cells were treated for four hours before harvesting. For the lipid pre-incubation experiment, a combination of 50 µM of each bile acid (i.e., a total of 150 µM of bile acids) and 12 mg or 16 mg of lipid mix [cholesterol 60–80 mg/g and protein 600–800 mg/g (Sigma-Aldrich, St. Louis, MO)] were pre-incubated at 37°C for 30 minutes before mixing with the media for a further four-hour incubation at 37°C. Bile acids and the lipid mix were diluted to the final concentration with water, as recommended by the manufacturer (Sigma-Aldrich, St Louis, MO). Pre-incubation was performed in the cell culture media (5 mL) before transferring 1.5 mL into 12–well plates.

The following liquid meal replacement options were tested for their effect on FGF19: Ensure Complete vanilla flavor, Ensure Clear peach flavor, Glucerna vanilla flavor (Abbott Park, IL), and Slimfast vanilla shake (Unilever, Englewood Cliffs, NJ). These meal replacement liquids were tested at 10% concentration (vol/vol) by diluting in cell culture media, pH 7.2. Powdered non-fat milk 5% (wt/vol) and whole milk 10% (vol/vol) were purchased at a local grocery store and also diluted to the final concentration by using cell culture media.

Caffeinated and decaffeinated instant coffees (Folgers Coffee Company, Orville, OH) were purchased from a local grocery store and used as 1% solutions (wt/vol). It was estimated that the 1% coffee solution contained 0.49 mg of caffeine. Higher concentrations of coffee (5% and 10%) were harmful to the cells. Caffeine was purchased from Sigma-Aldrich (St Louis, MO). The first concentration of caffeine used was identical to the one present in the 1% solution of instant coffee (i.e., 0.49 mg). The second concentration of caffeine used contained 10 times this amount (i.e., 4.9 mg of caffeine).

5-hour Energy pomegranate flavored drink (Living Essentials, Farmington Hills, MI) was purchased from a local grocery store and was used as a 10% solution (vol/vol).

The data are based on the mean value of a minimum of two experiments, each experiment performed in triplicates (in 1.5 mL well per replicate).

### FGF19 mRNA Levels by Real Time qPCR

FGF19 expression levels were measured by using the real time quantitative polymerase chain reaction (qPCR) method. Total RNA from cells was prepared by using the Total RNA Kit I (Omega, Norcross, GA). RNA quantitation was performed using a Nanodrop ND-1000 spectrophotometer (Thermo Scientific, Wilmington, DE).

RNA was reverse transcribed using a high capacity cDNA reverse transcription with RNase inhibitor kit (Applied Biosystems-Life Technologies, Carlsbad, CA). Quantitative PCR was performed in duplicate on the ABI 7500 Fast Plate (Applied Biosystems). Pre-designed assay for FGF19 was obtained from Applied Biosystems (Hs00192780_m1). GAPDH (Hs02758991_m1) was used as the calibrator gene. Analysis was conducted by subtracting the Ct value of GAPDH from the Ct value of the gene of interest. This delta value was adjusted by subtracting from 40, as previously described [20], so that gene downregulation does not appear with a negative sign but rather as a shorter column, relative to control, in the bar graphs.

### FGF19 Protein Levels

Cell media was collected after four hours of treatment and centrifuged to clear it of any cellular debris. The media was then centrifuged through a Millipore Centrifugal Filter Unit (Millipore, Billerica, MA) to concentrate the protein to 200 ul. Secreted FGF19 protein levels (pg/mL) was measured by using an Elisa kit according to the manufacturer’s instructions (R&D Systems, Minneapolis, MN) with sample, controls, and standards assayed in duplicate, as we have previously described [15]. This immunoassay employed the quantitative sandwich enzyme technique which is based on a monoclonal antibody specific to the human FGF19. The range of detection is 31–554 (pg/mL) with a standard deviation of 125 pg/mL. The coefficient of variation (CV) values of intra-assay and inter-assay precision are 4.5% and 5.5%, respectively.

### Statistical Analysis

Analysis of qPCR data was performed by one way analysis of variance (ANOVA) followed by Tukey’s test. Pairwise comparisons against the control were performed by the Students T-test All analyses were performed by using JMP Pro 10 (SAS Institute, Cary, NC), with the significance level set at P-value <0.05.

## Results

### Bile Acids Stimulate FGF19 mRNA and Secreted Protein Levels

**Figure 1:**
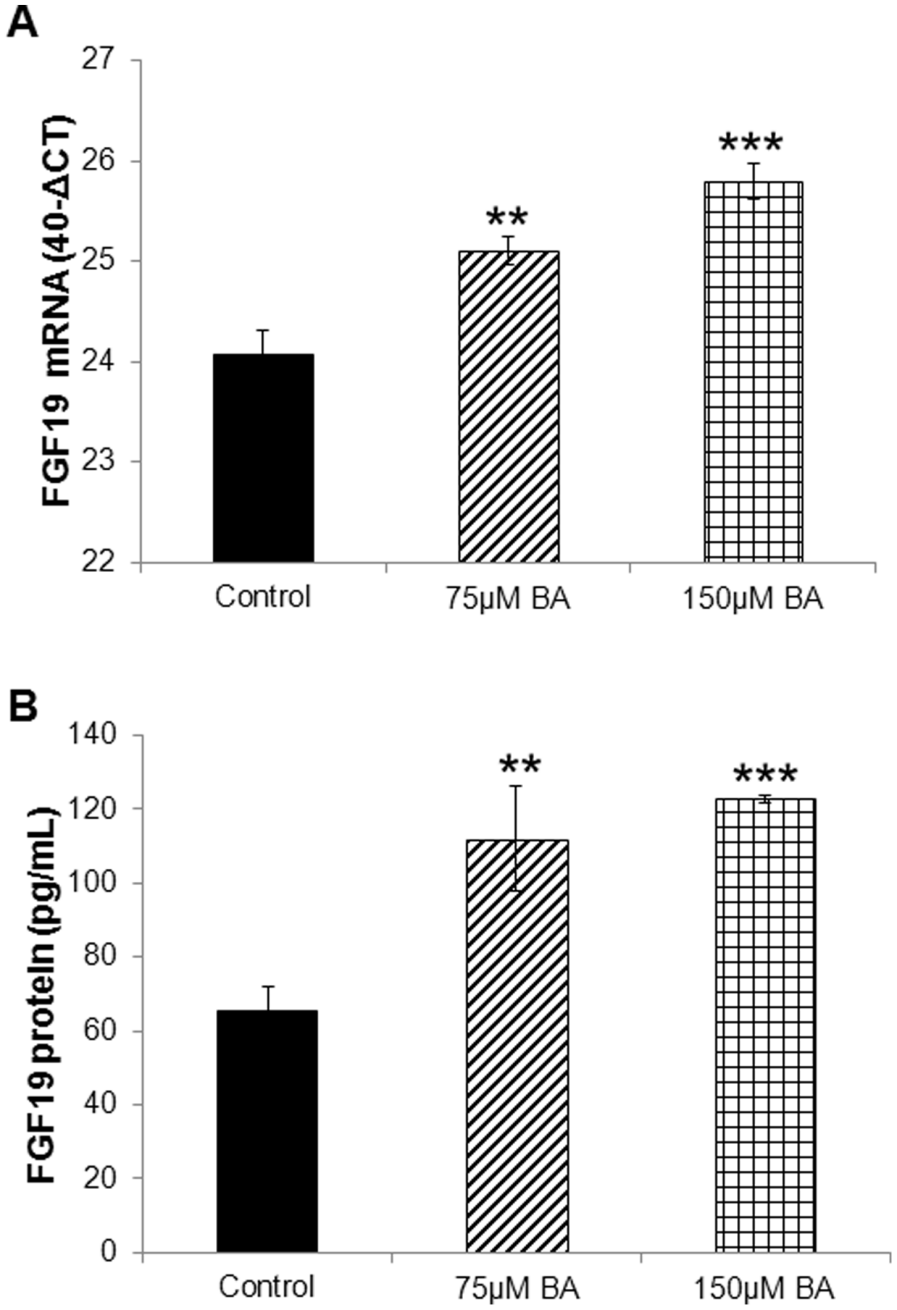
Bile acids stimulate FGF19 expression. (**A**) Increasing concentration of total bile acids proportionally increased FGF19 mRNA levels in LS174T cells after a 4-hour incubation. (**B**) Similarly, increasing concentration of total bile acids proportionally increased FGF19 protein levels in LS174T cells after a 4-hour incubation. (Comparisons were performed by one way ANOVA followed by Tukey’s test against the control, **: P<0.01, ***: P<0.001).

Bile acids were initially used in a cocktail of equimolar concentrations of unconjugated cholic acid (CA), deoxycholic acid (DA), and chenodeoxycholic acid (CDCA). Increasing concentrations of the combined bile acids (BA) proportionally increased endogenous expression of FGF19 mRNA (Figure 1A) and protein (Figure 1B) levels in the human colorectal LS174T cells.

**Figure 2:**
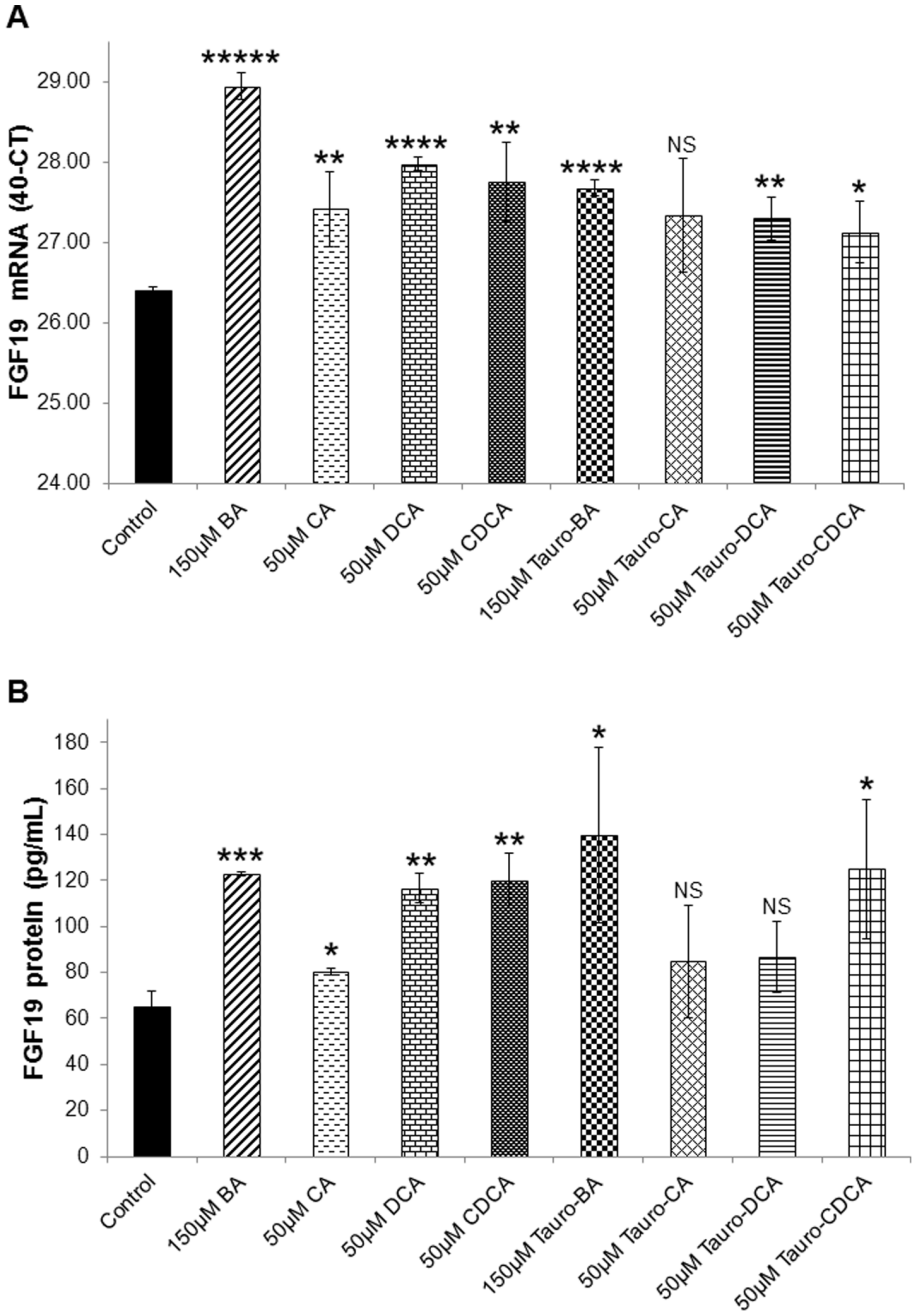
Effects of unconjugated and Tauro-conjugated bile acids on FGF19 expression. (**A**) Total bile acids had a more potent effect on FGF19 mRNA levels in LS174T cells after a 4-hour incubation, compared to cholic (CA), deoxycholic (DCA), chenodeoxycholic (CDCA) acids, and the corresponding tauro-conjugated bile acids. Individual bile acids also significantly stimulated FGF19 mRNA. (**B**) An analogous but less potent effect was observed on FGF19 protein levels under identical cell culture conditions. Pairwise comparisons against the control were performed by the Student’s T-test (*: P<0.05, **: P<0.01, ***: P<0.001, ****: P<0.0001, *****: P<0.00001).

Total and individual unconjugated and tauro-conjugated bile acids were used at the concentration of 50 µM to compare their effects on FGF19 expression. Both mRNA (**Figure 2A**) and secreted protein (Figure 2B) levels were stimulated by most BA. The unconjugated BA (total or individual), however, had a more potent effect at both the mRNA and protein levels, compared to the tauro-conjugated BA (**Figure 2** **A&B**).

Glycine-conjugated CA, glycine-DCA, and glycine-CDCA were also used but they did not have a significant effect on FGF19 expression in the LS174T cells after a 4-hour treatment, either singularly or when mixed together.

### Pre-incubation of Bile Acids with Lipoproteins Diminishes the Stimulation of FGF19 mRNA

**Figure 3:**
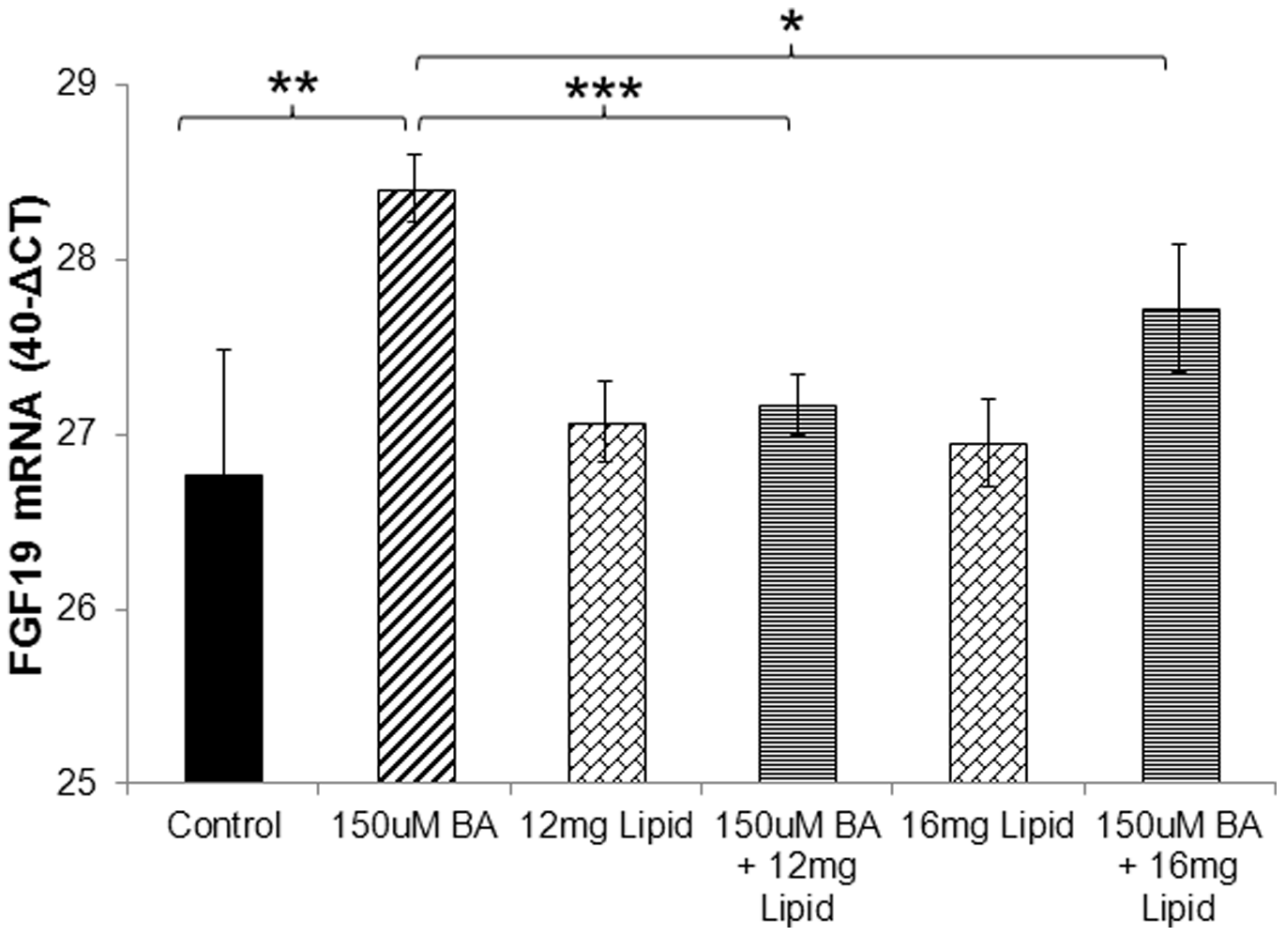
Pre-incubation of bile acids with a lipid mix attenuates the stimulation of FGF19. The combined unconjugated bile acids (cholic, deoxycholic, and chenodeoxycholic) were pre-incubated for 30 minutes with two different concentrations of lipid mix (consisting of cholesterol and protein) prior to addition in cell culture for 4 hours. Increasing amounts of the lipid mix proportionally attenuated the stimulatory effects of bile acids on FGF19 mRNA. Pairwise comparisons against the control or the 150 µM BA treatment were performed by the Student’s T-test (*: P<0.05, **: P<0.01, ***: P<0.001).

The treatments of LS174T cells (**Figures 1** **&** **2**) showed that both mRNA and protein levels of FGF19 were increased in an analogous fashion and corresponding to the dose of BA. Hence, subsequent experiments were performed by measuring only mRNA levels. Bile acids were pre-incubated with a lipid mix consisting of cholesterol and protein for 30 minutes prior to addition in cell culture for the routine 4-hour treatment. The lipid mix by itself had no effect on endogenous FGF19 mRNA in LS174T cells. The stimulatory effects of bile acids on FGF19, however, were significantly diminished by the pre-incubation with two different concentrations of the lipid mix (**Figure 3**).

### Effects of Meal Replacement Drinks, Coffee, Milk, and an Energy Drink on FGF19 mRNA

In order to determine whether prepackaged nutrients could stimulate FGF19 like BA do, we used the meal replacement liquids Ensure Complete, Ensure Clear, Glucerna, SlimFast, whole milk, non-fat milk, caffeinated and decaffeinated instant coffee, and the 5-hr energy drink, all purchased at a local grocery store. All meal replacement liquids, whole milk, and 5-hr energy were used as a final 10% solution (vol/vol) (Table 1). Non-fat milk was used as a 5% solution (vol/vol) while instant coffee was used as a 1% solution (wt/vol).

**Table 1:** Summary of contents of meal replacement liquids, milk, coffee, and 5-hr energy drink.

	EnsureComplete	EnsureClear	Glucerna	SlimFast	Whole milk	Non-fat milk	Caf Coffee	Decaf Coffee	5-hr energy
**Final concentration**	10%	10%	10%	10%	10%	5%	1%	1%	10%
**Calories**	0.22	0.09	0.13	0.09	0.13	0.26	0	0	0
**Fat**	6.97	0	4.44	3.04	5.07	0	0	0	0
**Saturated Fat**	0.63	0	0.32	0.76	2.85	0	0	0	0
**Trans Fat**	0	0	0	0	0	0	0	0	0
**Cholesterol**	0.0032	0.0025	<0.0032	0.0051	0.0159	<0.0163	0	0	0
**Sodium**	0.15	0.0254	0.13	0.11	0.0602	0.41	0	0	0.0473
**Potassium**	0.36	0.0228	0.24	0.28	0	1.27	0	0	0
**Carbohydrate**	32.33	17.75	16.48	12.17	6.97	39.13	0	0	0
**Dietary Fiber**	1.90	0	1.90	2.54	0	0	0	0	0
**Sugars**	12.68	9.13	3.80	9.13	6.97	39.13	0	0	0
**Protein**	8.24	4.56	6.34	5.07	5.07	26.09	0	0	0
**Caffeine**	NA	NA	NA	NA	NA	NA	0.49	0.0225	NA
**Energy blend** ¶	NA	NA	NA	NA	NA	NA	NA	NA	4.91

The indicated percentages are based on the label of each product and refer to the final concentration (percent - %) as used during the treatment of LS174T cells. NA: not available on the product label. All units (except for the “percent” - % - concentration and “Kcal” for calories) represent “mg”.

Energy blend: taurine, glucuronic acid, malic acid, n-acetyl l- tyrosine, l-phenylalanine, caffeine, citicoline.

**Figure 4:**
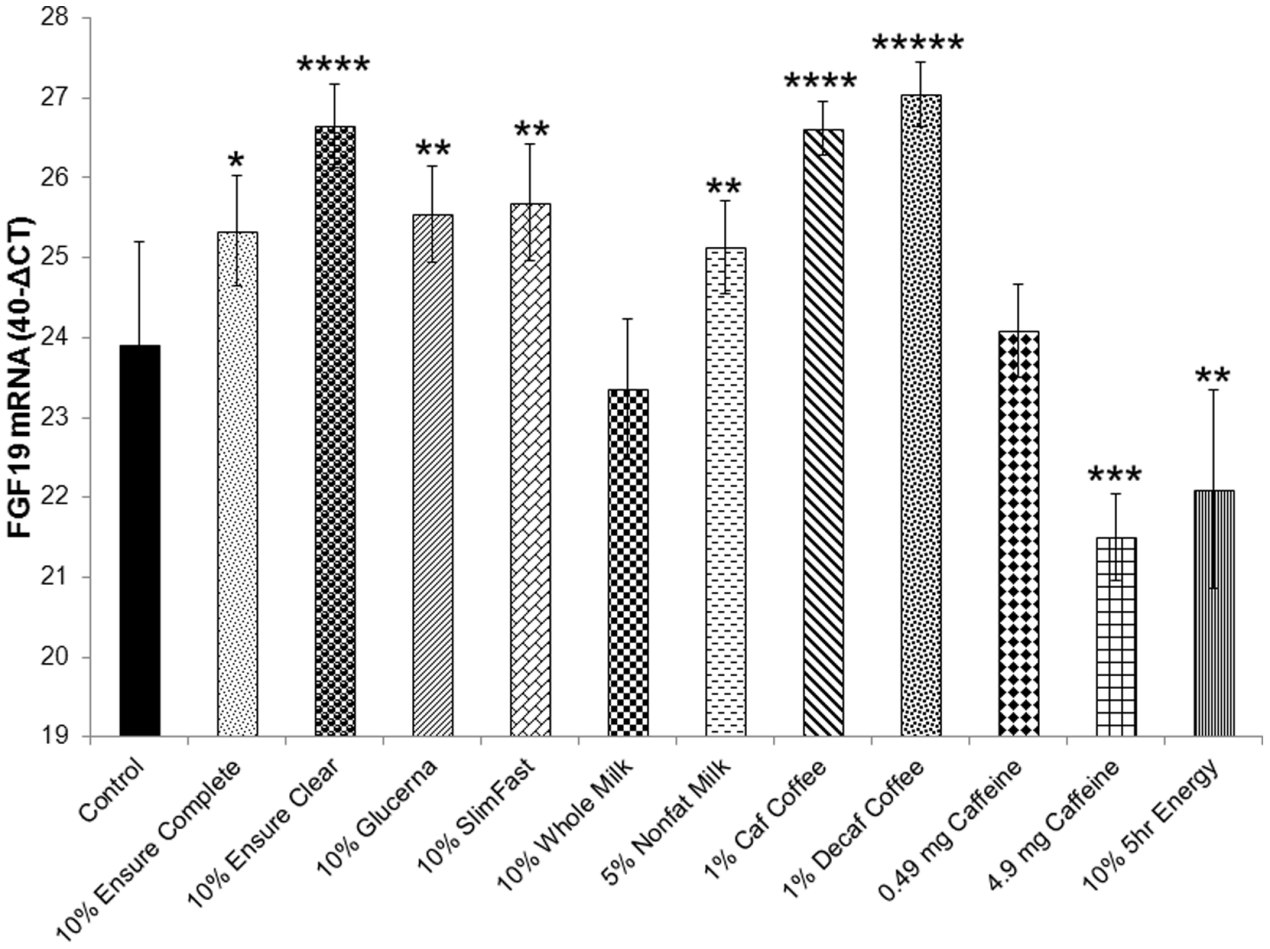
Effects of meal replacement drinks, milk, coffee, 5-hour energy, and pure caffeine, on FGF19 expression. Ensure complete, Ensure-clear, Glucerna, SlimFast, and non-fat milk, stimulated FGF19 mRNA in LS174T cells after a 4-hour treatment. Ensure-clear had the strongest effect. Whole milk had no effect. Coffee (both caffeinated and decaffeinated) stimulated FGF19 expression, while, high concentration of pure caffeine and the 5-hour energy drink and decreased FGF19 mRNA (pairwise comparisons by T-Test against the control, *: P<0.05, **: P<0.01, ***: P<0.001, ****: P<0.0001, *****: P<0.00001).

All meal replacement drinks, non-fat milk, and caffeinated coffee significantly stimulated FGF19 mRNA with decaffeinated coffee having the most profound effect (**Figure 4**). Whole milk had no significant effect on FGF19 expression (**Figure 4**). The 5-hour energy drink and pure caffeine solution significantly decreased FGF19 mRNA (**Figure 4**). 1% of caffeinated coffee solution contains 0.49 g of caffeine.

The various concentrations used in these experiments were chosen because they had the most significant effects without harming the cells. Higher and lower concentrations were also examined but were found to have lesser effects or to be harmful to the LS174T cells after 16-hour treatments (results not shown). Details regarding the contents of each solution, as used in its final concentration, are provided in **Table 1**.

## Discussion

The role of FGF19 in hepatic glucose homeostasis is under intense investigation [21]–[23]. We recently showed that FGF19 serum levels are decreased and bile acid levels increased in patients with type 2 diabetes [24]. Moreover, FGF19 and bile acids were found to increase predominantly in patients that experience diabetes remission after Roux-en-Y gastric bypass surgery, compared to patients without diabetes or patients with diabetes that do not experience remission after RYGB surgery [24]. This effect is hypothesized to be centered on the fact that after surgery digestate-free bile acids bypass ∼1.5 meters of the jejunum and are secreted near the ileum where FGF19 is produced. These digestate-free bile acids are expected to be more bioactive and thus have more profound effects on FGF19 stimulation.

First, we showed that equimolar combinations of unconjugated cholic, deoxycholic, and chenodeoxycholic acids could potently stimulate FGF19 expression (both mRNA and secreted protein), as previously reported in the same cells [10], [11] and in ileal explants [25]. When using individual, unconjugated bile acids, CDCA had the most potent and consistent stimulatory effect. Tauro-conjugated bile acids also stimulated significantly FGF19 expression (at both mRNA and protein levels) but to a lesser extent, compared to the unconjugated BA. When the cocktail of all unconjugated bile acids, however, was pre-incubated with a mix of cholesterol and protein, which could emulate the presence of digested food *in vivo*, its stimulatory effects were partially inhibited. This finding supports our hypothesis that digestate-free bile acids are likely more bioactive and thus could potentially have a stronger effect on enteric FGF19 stimulation after RYGB surgery [15]. Additional experiments (perhaps involving cleansing of the intestine) are needed to confirm this finding and its direct impact on the stimulation of FGF19 by BA in humans.

Furthermore, we examined the effects of popular meal replacement drinks on FGF19 mRNA to determine whether FGF19 could be stimulated by prepackaged nutrients in a fashion that is stimulated by BA. Customized diets of low calorie content have been used extensively and reported to improve glucose and HbA1C levels [26], [27] but the effects of meal replacements, milk, coffee, and energy drinks are not known. SlimFast and Glucerna have been used by participants of the Look AHEAD study but only as part of other sources of nutrient intake [28] and of unknown mechanistic actions. Our experiments showed that all popular meal replacement drinks (Ensure, Glucerna, SlimFast), along with non-fat milk, had a stimulatory effect on FGF19. Ensure Clear had the most potent effect and it should be noted that it was the only drink that did not contain any form of fat which is similar to non-fat milk which also had a potent stimulatory effect on FGF19 mRNA. We hypothesize that the absence of fat in these two drinks, and/or the presence of a yet-to-be determined ingredient may be responsible for the enhanced potency of the effect in Ensure clear and non-fat milk on FGF19 expression. Further experiments using individual components (e.g., protein alone, or fat alone, or carbohydrates alone) would need to be performed to address this question.

Caffeinated and decaffeinated coffee in particular, also had potent stimulatory effects on FGF19 expression. Using two different concentrations of caffeine, we showed that caffeine itself is unlikely to be the stimulatory ingredient. In fact, the 10-fold higher concentration of caffeine significantly attenuated FGF19 expression. It would therefore appear that the stimulatory effects of coffee are due to other ingredients in the coffee beans that, *in vivo*, have been shown to stimulate incretins [29], [30]. Consumption of coffee, and in particular decaffeinated coffee [31] have been consistently associated with reduced risk for developing type 2 diabetes [19], [32]. The analogous effects of decaffeinated coffee on the stimulation of FGF19 (which is associated with diabetes remission after RYGB) [15]and its reported association with reduced risk of diabetes could potentially be correlated. It would therefore be of interest to measure in the future FGF19 levels in humans consuming 4–8 cups of decaffeinated coffee and compare them to participants who are not consuming coffee.

The 5-hour energy drink was the only liquid (other than the high caffeine concentration) that downregulated FGF19 mRNA. This could be due to the potential presence of stimulants like caffeine which are not specified on the label of the product, or other components like niacin, vitamin B6, or folic acid that are listed on the label.

In summary, meal replacement drinks (particularly fat-free), non-fat milk, and coffee (both decaf and caffeinated) had powerful stimulatory effects on FGF19 expression. This could potentially be beneficial in reducing risk for diabetes development because higher FGF19 levels are associated with non-diabetes and diabetes remission after RYGB surgery [15]. High caffeine concentration and the 5-hour energy drink, on the other hand, had negative effects on FGF19 expression. These data should be corroborated by *in vivo* studies before any conclusions can be made regarding the beneficial (or detrimental) effects of these popular drinks towards FGF19 expression and their potential effects with respect to the development of diabetes in humans.
